# Host–symbiont combinations dictate the photo-physiological response of reef-building corals to thermal stress

**DOI:** 10.1038/s41598-019-46412-4

**Published:** 2019-07-10

**Authors:** Kenneth D. Hoadley, Allison M. Lewis, Drew C. Wham, D. Tye Pettay, Chris Grasso, Robin Smith, Dustin W. Kemp, Todd C. LaJeunesse, Mark E. Warner

**Affiliations:** 1 School of Marine Science and Policy, University of Delaware, Lewes, DE United States; 2 Department of Biology, Pennsylvania State University, University park, PA United States; 3 Department of Biology, University of Alabama at Birmingham, Birmingham, AL United States; 4 Science Under Sail, Sarasota, United States; 5 Present Address: GEOMAR Helmholtz Centre for Ocean Research, Kiel, Germany

**Keywords:** Marine biology, Climate-change ecology

## Abstract

High sea surface temperatures often lead to coral bleaching wherein reef-building corals lose significant numbers of their endosymbiotic dinoflagellates (Symbiodiniaceae). These increasingly frequent bleaching events often result in large scale coral mortality, thereby devasting reef systems throughout the world. The reef habitats surrounding Palau are ideal for investigating coral responses to climate perturbation, where many inshore bays are subject to higher water temperature as compared with offshore barrier reefs. We examined fourteen physiological traits in response to high temperature across various symbiotic dinoflagellates in four common Pacific coral species, *Acropora muricata*, *Coelastrea aspera*, *Cyphastrea chalcidicum* and *Pachyseris rugosa* found in both offshore and inshore habitats. Inshore corals were dominated by a single homogenous population of the stress tolerant symbiont *Durusdinium trenchii*, yet symbiont thermal response and physiology differed significantly across coral species. In contrast, offshore corals harbored specific species of *Cladocopium* spp. (ITS2 rDNA type-C) yet all experienced similar patterns of photoinactivation and symbiont loss when heated. Additionally, cell volume and light absorption properties increased in heated *Cladocopium* spp., leading to a greater loss in photo-regulation. While inshore coral temperature response was consistently muted relative to their offshore counterparts, high physiological variability in *D*. *trenchii* across inshore corals suggests that bleaching resilience among even the most stress tolerant symbionts is still heavily influenced by their host environment.

## Introduction

The mutualistic symbioses between scleractinian corals and their endosymbiotic dinoflagellates, Symbiodiniaceae, is critical to the continual growth of coral reef systems. High genetic diversity within both the host and symbiont provides a remarkable amount of variability in coral phenotypes and will likely lead to differences in their response to future climate change. Indeed, corals exhibit differences in bleaching, a now common occurrence, wherein high temperature anomalies lead to a significant disruption in this mutualistic symbioses and significant losses of these intracellular algae. Measures of photobiology, such as active chlorophyll *a* fluorescence, have helped elucidate a number of key break points in the photosynthetic electron transport chain as well as stress mitigating pathways across different species, which have helped identity thermally tolerant algal phenotypes^1–4^. However, physiological differences across host species also affect the *in hospite* (internal) endosymbiotic environment, potentially influencing symbiont susceptibility to thermal stress^5–7^. Hence, a better functional understanding of how symbiont physiology corresponds to the host species that they inhabit is necessary to help identify thermally resilient coral phenotypes.

In general, the Rock Island habitats of Palau have higher water temperatures (~2 °C) and lower pH (~0.15 pH) than the offshore habitats^8–11^. Given current climate change projections, conditions similar to those found within the Rock Islands are expected for many tropical reef systems^12^. Thus, Palauan reefs are a good comparative system for studying possible effects of climate change on the potentially unique physiology of many different host and symbiont combinations. Historically, corals from inshore habitats in Palau bleach less than conspecifics living on offshore barrier reefs^10,13^.

Following major high temperature anomalies in 1998 and 2010, Rock Island corals experienced lower mortality and greater bleaching recovery rates^10,13^. As climate projections suggest a continued increase in seasonal water temperatures and more frequent bleaching events^12,14^, efforts are underway to understand the biology of reef corals in inshore and offshore habitats^11,15^. Some of this resistance to thermal stress could be attributed to the dominance of a thermally tolerant endosymbiont, *Durusdinium trenchii*, among inshore corals^8^. Similar differences in symbiont species exist across environmental gradients for some Pacific and Persian/Arabian Gulf corals and are essential in establishing high and low thermal tolerance^16–18^.

Apart from differences in symbiont composition, previous studies of inshore reefs in Palau have attributed bleaching resistance to light attenuation in highly turbid water and physical shading provided by the thick vegetation bordering Rock Island reefs^8,10^. Indeed, exposure to high light exacerbates thermal stress among certain coral species, as excess excitation energy increases susceptibility to thermal photoinactivation in some symbionts^19,20^. Resistance to thermal stress may also correspond to local inshore acclimatization or a higher proportion of adapted coral genotypes that have successfully proliferated over many generations^21,22^.

Host coral biology influences resistance to bleaching in many ways^5–7^, including tissue thickness and structural differences in the underlying CaCO_3_ skeleton^3,23–26^, that substantially influence *in hospite* light fields^27^. Additionally, host derived photoprotective pigments may further affect internal light fields^28–30^, whereas different mechanisms for scavenging reactive oxygen species (ROS), along with heat-shock protein expression, can mitigate damage regardless of the resident symbiont species^31^. Heterotrophically derived higher energy reserves and/or nutrients^32–35^ may also improve resilience and recovery to thermal stress. In addition, transcriptional differences in stress response genetic pathways also play critical roles in establishing thermal tolerance^36–39^.

While field-based evidence suggests greater bleaching resistance in corals surrounding the Rock Islands, the mechanisms for such thermal tolerance are unknown. Most Indo-Pacific corals must acquire their dinoflagellates from the environment at the beginning of each new generation (i.e. ‘open’ systems). The habitat in which larvae settle often dictates which kind of host-generalist alga will occupy the adult colony^40,41^. Such flexibility in host-symbiont compatibility contributes to the ecological success of coral communities^17,40–42^. Inshore coral species with open modes of symbiont acquisition are often dominated by the stress-tolerant *Durusdinium trenchii*^8,43–45^.

We examined the relationship between symbiont identity to coral stress response in four coral species from inshore Rock Island and offshore barrier reef habitats during a short-term exposure to high temperature (32 °C). A range of symbiont specific photobiological and biochemical proxies were used to characterize phenotypic response, which was then compared to the genetic variation in *D*. *trenchii* populations and offshore *Cladocopium* (formerly Clade C) symbionts. We noted several similar proxies for thermal photoinactivation in offshore corals harboring different species of *Cladocopium*, while inshore corals harboring *D*. *trenchii* displayed a range of functional responses that were influenced by their host coral species. This study advances our understanding of how symbiont physiology can be substantially modulated when living in different hosts.

## Results

### Symbiont species identification, genotyping and population genetic structure

**Figure 1:**
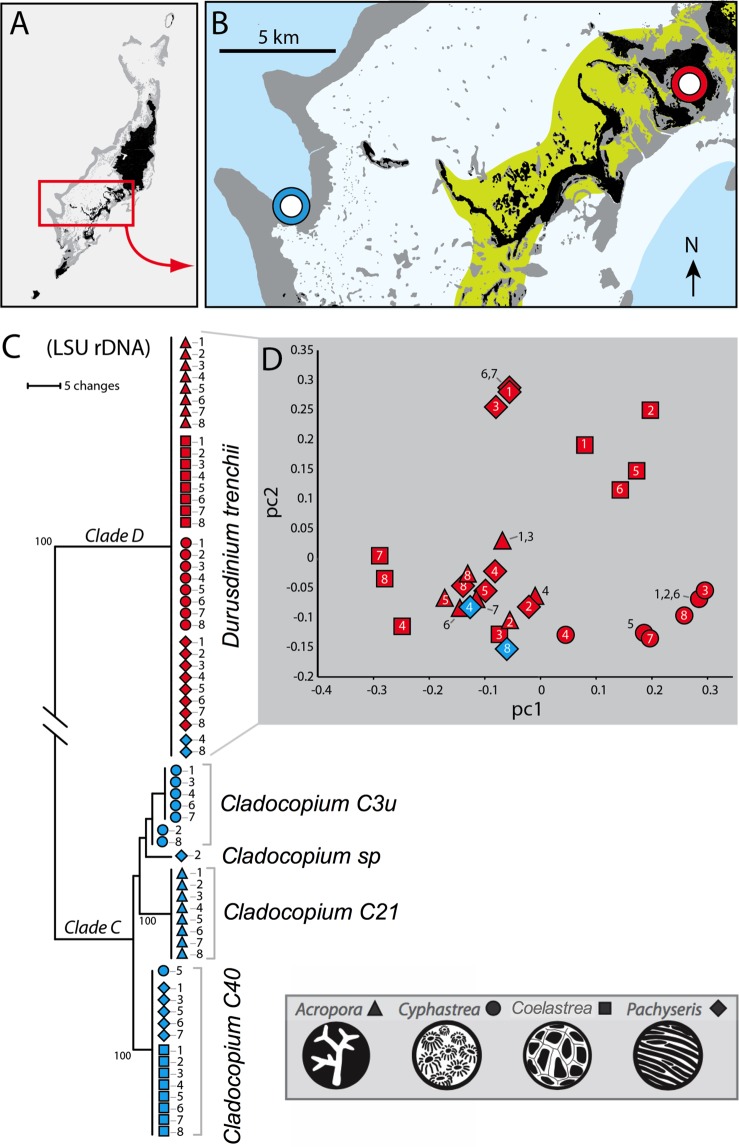
Map of study sites and genotypic analysis for *D*. *trenchii* symbionts. The map (panel A,B) reflects the sites across Palau where coral colonies were collected. The blue and red circles in panel B reflect the Offshore and Nikko Bay sampling sites respectively. DNA phylogeny (panel C) based on the Ribosomal large sub-unit (LSU) depicts symbiont identities among Offshore (Blue) and Nikko Bay (Red) collects coral colonies. Bootstrap values based on 1000 iterations are indicated below branch lengths. Principal coordinate analysis (panel D) of population genetic data on *Durusdinium trenchii* genotypes (based on 14 microsatellites) shows considerable genetic variation and minimal clonality among experimental colonies. The different shapes in panel C and D reflect coral species (*A*. *muricata* = triangle; *C*. *chalcidicum* = circle, *C*. *aspera* = square; *P*. *rugosa* = diamond). Accompanying numbers adjacent to each shape reflect species colony number.

Only *Durusdinium trenchii* was identified (by ITS2 screening and LSU rDNA sequencing) in all corals from inshore habitats (Fig. 1C). Further characterization of distinct multilocus genotypes (i.e. clones) of *D*. *trenchii* by 14 microsatellite loci identified a single strain dominated each colony (controls and treatments), but were typically different from colony to colony. Genetic variance of *D*. *trenchii* multilocus genotypes were visualized using a principal coordinate analysis, whereas the statistical analysis were performed using the program GenAIEx (Fig. 1D). Analysis of molecular variance (AMOVA) also suggested that some clustering of variance was attributed to the host coral species (e.g. *C*. *chalcidicum*). However, only 9% of the overall population variance was attributed to host species, while inter individual algal variance accounted for the remaining 91% of the variation across samples. When the analysis also included additional samples collected in 2009, an even greater variance for *D*. *trenchii* genotypes in *C*. *chalcidicum* was noted than was observed for experimental colonies alone (Supplementary Fig. 1). For offshore corals, both *C*. *aspera* and *P*. *rugosa* harbored *Cladocopium C40* whereas *A*. *muricata* harbored *Cladocopium C*21 and *C*. *chalcidicum* harbored *Cladocopium 3u* (Fig. 1C). The dominant symbiont did not change in any coral or treatment throughout the duration of the experiment (day 0, 9 and 14). However, mixed assemblages of *D*. *trenchii* and *Cladocopium C40* were noted in 2 colonies of offshore *P*. *rugosa*. Likewise, *Cladocopium C40* was also found in a single colony of *C*. *chalcidicum*. These colonies were removed from further analyses described below.

### Differences in bleaching responses among offshore and inshore corals

**Figure 2:**
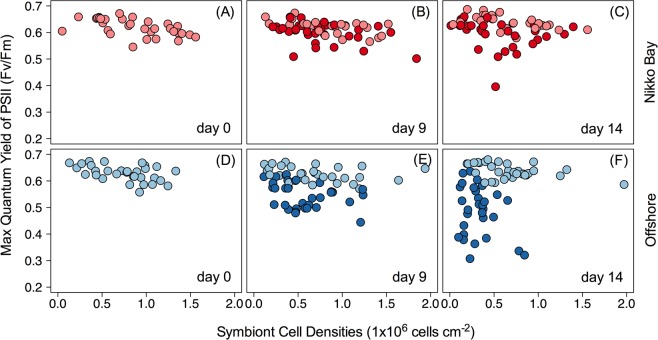
Temporal response to elevated temperature. 2-dimensional plots of cellular density (1 × 10^6^ cells cm^−2^) and maximum quantum yield of PSII (FvFm) by PAM fluorometry throughout the 14-day experiment. Left: initial time point for Nikko Bay (**A**) and offshore (**D**) corals. Middle: day nine of the experiment (four days of ramping followed by 5 days at 32 °C) for Nikko Bay (**B**) and Offshore (**E**) corals. Right: final day of the experiment (four days of ramping followed by ten days at 32 °C) for Nikko Bay (**C**) and Offshore (**F**) corals. Light colors indicate (27.5 °C) and dark colors indicate (32 °C).

While there was significant separation in photochemistry and symbiont number between ambient and high temperature treated inshore corals on day 14 (4-day ramping + 10 days at 32 °C) (*P* = 0.0198), the ANOSIM R-value was less than 0.1 (R = 0.066), suggesting minimal change (Fig. 2, Table 2). In contrast, separation between control and heated offshore corals was significant on both day 9 (4 days of ramp-up and 5 at 32 °C) (ANOSIM: R = 0.160, *P* = 3e-04) and day 14 (ANOSIM: R = 0.482, *P* = 1e-04) (Fig. 2, Table 2). Univariate analyses of offshore corals noted a small but significant reduction in *F*_v_/*F*_m_ (*P* < 0.001) on day 9 and significant loss in symbiont density (*P* < 0.0001) and *F*_v_/*F*_m_ (*P* < 0.0001) on day 14. For Inshore corals, only *F*_v_/*F*_m_ was significantly reduced on day14 at 32 °C (*P* < 0.0001), and this trend was driven largely by *P*. *rugosa* (Table 2).

**Table 1:** Table of terms, definitions and units.

Term	Definition	Units
*F*_v_/*F*_m_^MT^	Dark acclimated maximum quantum yield of PSII (multi-turnover)	relative units
σ_PSII_	Dark acclimated effective absorption cross section of PSII	Å^2^q^−1^
τ_PSII_	Rate constant for reoxidation of the Q^a^ site of the D1 protein within the PSII RC	μ-seconds
τ_PQ_	Rate constant for reoxidation of the plastoquinole pool.	μ-seconds
ETR	RCII-specific electron transport rate	mol e^−^ mol RCII^−1^h^−1^
NPQ	Non-photochemical quenching	relative units
P_gross_	gross algal photosynthesis	mg O_2_ L^−1^ min^−1^ cell^−1^
ρ	Connectivity between PSII reaction centers	relative units
Carbohydrates	Carbohydrate concentration cell^−1^	μg cell^−1^
Protein	Protein concentration cell^−1^	μg cell^−1^
Lipids	Lipid concentration cell^−1^	μg cell^−1^
Chlorophyll	Chlorophyll concentration cell^−1^	pg cell^−1^
Cell Volume	Symbiont cellular volume	μm^−3^
Cell Density	Symbiont number normalized to coral area	Cells cm^−2^

**Table 2:** Overall bleaching response per habitat. ANalysis Of SIMilarity (ANOSIM with 9,999 permutations), for day 9 and day 14 for Offshore and Nikko bay corals. Wilcox test for each variable follow multivariate analysis with R > 0.05.

Location	Source of variation	Time	ANOSIM		Univariate Wilcox Test (P-value)	
			R	*P* value	Density	FvFm
Offshore	Temperature	Day 9	**0**.**160**	**3e-04**	0.4588	**2**.**7e-09**
	**Temperature**	**Day 14**	**0**.**482**	**1e-04**	**1**.**48e-06**	**1**.**7e-10**
Nikko Bay	Temperature	Day 9	0.028	0.1077		
	**Temperature**	**Day 14**	**0**.**066**	**0**.**0198**	0.1402	**4**.**06e-05**

### Endpoint measurements (Day 14)

**Figure 3:**
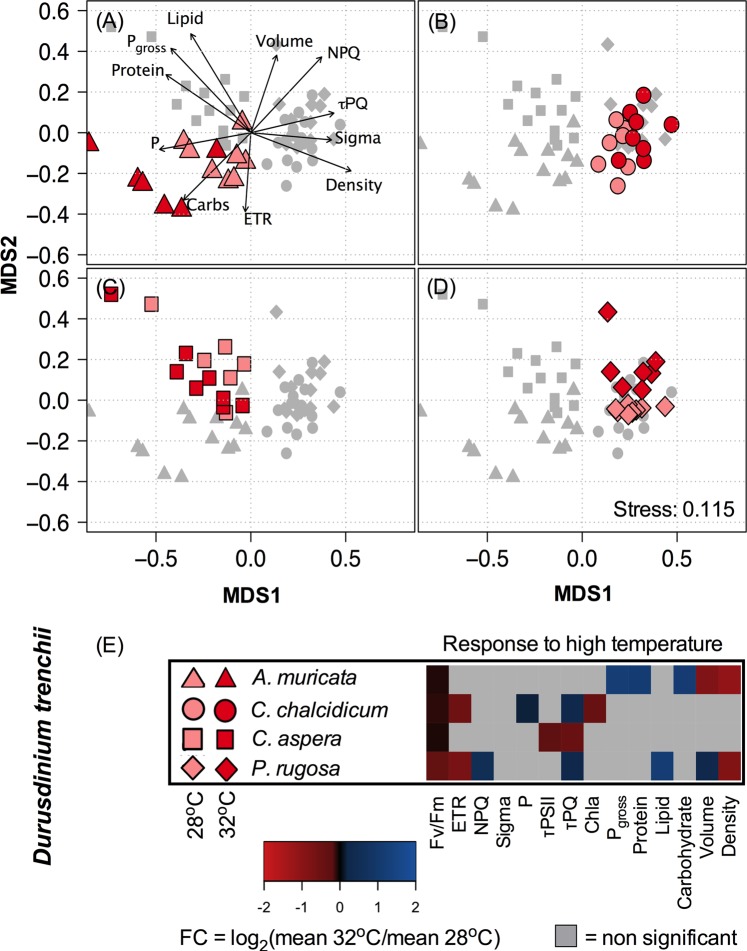
Differential analysis for *D*. *trenchii* symbioses in Nikko Bay corals (Inner Reef). The resulting MDS for *D*. *trenchii* is plotted with individuals from different host species highlighted within each panel (A) *A*. *muricata*, (B) *C*. *chalcidicum*, (C) *C*. *aspera* and (D) *P*. *rugosa*. Light vs. dark red reflect ambient (27.5 °C) and elevated (32 °C) temperature treatments respectively while shapes depict the host coral species (circle) *C*. *chalcidicum*, (triangle) *A*. *muricata*, (square), *C*. *aspera*, (diamond) *P*. *rugosa*. Physiological parameters are represented through vectors with significant (*P* < 0.001, 999 permutations) correlation to the plotted sample points (represented in panel A only). For each coral species, the heat map (**E**) reflects the average Log^2^ fold change (FC) in response to elevated temperature for each physiological variable. Variables where no significant change in response to temperature was observed are displayed in grey. All physiological variables are described in Table 1 and average values can be found in Supplemental Table 1.

Physiological responses of *Durusdinium trenchii* separated significantly across all inshore corals held at the control (ambient) temperature (ANOSIM: R > 0.605, *P* < 0.001, Table 3, Fig. 3).

**Figure 4:**
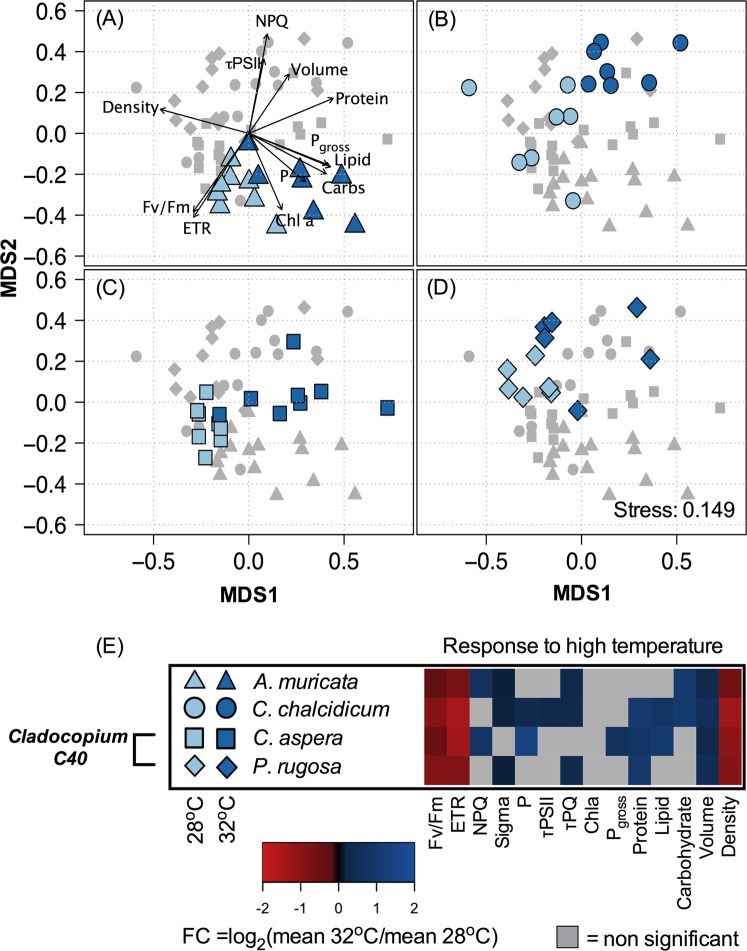
Differential analysis for Offshore *Cladocopium* symbioses (Outer Reef). The resulting MDS for offshore *Cladocopium* symbioses is plotted with individuals from different host species highlighted within each panel (A) *A*. *muricata*, (B) *C*. *chalcidicum*, (C) *C*. *aspera* and (D) *P*. *rugosa*. Light vs. dark blue reflect ambient (27.5 °C) and elevated (32 °C) temperature treatments respectively while shapes depict the host coral species (circle) *C*. *chalcidicum*, (triangle) *A*. *muricata*, (square), *C*. *aspera*, (diamond) *P*. *rugosa*. Physiological parameters are represented through vectors with significant (*P* < 0.001, 999 permutations) correlation to the plotted sample points (represented in panel A only). For each coral species, the heat map (**E**) reflects the average Log^2^ fold change (FC) in response to elevated temperature for each physiological variable. Variables where no significant change in response to temperature was observed are displayed in grey. All physiological variables are described in Table 1 and average values can be found in Supplemental Table 1.

Similarly, symbiont physiology differed significantly between *Cladocopium C40* symbionts in offshore *Coelastrea aspera* and *Pachyseris rugosa* (ANOSIM: R = 0.793, *P* = 6e-04) (Table 3, Fig. 4). MDS plots provide a visualization of physiological differences in symbionts across inshore (Fig. 3) and offshore (Fig. 4) corals. Vectors in each MDS plot (panel A of each plot) represent specific parameters that contributed significantly to the ordination (*P* < *0*.*001*, 999 permutations), and plotted points are maximally correlated to these vectors and provide insight into how they drove the observed differences. For example, the orientation of the τ_PQ_ vector (Fig. 3A) demonstrates that plastoquinone pool reoxidation values are higher (i.e. slower reoxidation rates) within *C*. *chalcidicum* and *P*. *rugosa* as compared with *A*. *muricata* and *C*. *aspera* (compare Fig. 3B,D to A,C). Vectors for protein and lipids indicate highest concentrations for symbionts within the host coral *C*. *aspera* whereas the carbohydrate vector indicates highest concentrations within high temperature symbionts in *A*. *muricata* (Fig. 3A–D).

**Table 3:** Comparison of symbiont physiology across hosts and temperature treatments. ANalysis Of SIMilarity (ANOSIM with 9,999 permutations), for day 14. ANOSIM for each host/symbiont combination in response to temperature (Top). Comparison of ambient temperature symbiont physiology within hosts found to contain the same symbiont type (Bottom).

	Source of variation	Host Coral	R	*p* value
***D. trenchii***	Temperature	***A***. ***muricata***	**0**.**650**	**0**.**0022**
***D. trenchii***	Temperature	*C*. *aspera*	0.169	0.0768
***D. trenchii***	Temperature	***C***. ***chalcidicum***	**0**.**219**	**0**.**0378**
***D. trenchii***	Temperature	***P***. ***rugosa***	**0**.**669**	**2e-04**
***C. 40***	Temperature	***C***. ***aspera***	**0**.**687**	**2e-04**
***C. 21***	Temperature	***A***. ***muricata***	**0**.**633**	**5e-04**
***C. 3u***	Temperature	***C***. ***chalcidicum***	**0**.**782**	**6e-04**
***C. 40***	Temperature	***P***. ***rugosa***	**0**.**613**	**0**.**0026**
***D. trenchii***	Host species	***A***. ***muricata – C***. ***aspera***	**0**.**798**	**5e-04**
***D. trenchii***	Host species	***A***. ***muricata – C***. ***chalcidicum***	**0**.**847**	**2e-04**
***D. trenchii***	Host species	***A***. ***muricata – P***. ***rugosa***	**0**.**944**	**4e-04**
***D. trenchii***	Host species	***C***. ***aspera – C***. ***chalcidicum***	**0**.**870**	**5e-04**
***D. trenchii***	Host species	***C***. ***aspera – P***. ***rugosa***	**0**.**920**	**3e-04**
***D. trenchii***	Host species	***C***. ***chalcidicum – P***. ***rugosa***	**0**.**605**	**2e-04**
***C. 40***	Host species	***C***. ***aspera – P***. ***rugosa***	**0**.**793**	**6e-04**

*Durusdinium trenchii* response to heating within inshore corals varied substantially across host/symbiont combinations and resulted in significant separation by treatment in *Acropora muricata* (ANOSIM: R = 0.650, *P* = 0.0022), *Pachyseris rugosa* (ANOSIM: R = 0.669, *P* = 0.0002) and *Cyphastrea chalcidicum* (ANOSIM: R = 0.219, *P* = 0.0378) (Table 3). However, there was no significant separation between ambient and elevated temperature samples in *Coelastrea aspera* (ANOSIM: R = 0.169, *P* = 0.0768) (Table 3). Notably, for each coral, response to elevated temperature was unique (Fig. 3A–D). For *A*. *muricata*, algal soluble protein, carbohydrate, and net photosynthesis cell^−1^ rose significantly with temperature, whereas *F*_v_/*F*_m_^MT^, algal density and algal cell volume significantly declined (Fig. 3E, Table S3). NPQ, the time constant for PQ reoxidation (τ_PQ_), cellular lipid and volume all increased with high temperature in *D*. *trenchii* within *P*. *rugosa*. Meanwhile, ETR, symbiont density and *F*_v_/*F*_m_^MT^ all decreased (Fig. 3E, Table S4). The time constant for PSII reoxidation (τ_PSII_), *F*_v_/*F*_m_^MT^ and τ_PQ_ all decreased in *D*. *trenchii* in *C*. *aspera* under elevated temperature (Fig. 3E, Table S5). Lastly, elevated temperature resulted in a significant drop in *F*_v_/*F*_m_^MT^, ETR and Chl *a* for *D*. *trenchii* in *C*. *chalcidicum*. In contrast, τ_PQ_, along with PSII connectivity (ρ), increased under elevated temperature (Fig. 3E, Table S6).

Similar to inshore corals, physiological responses in the offshore corals separated significantly with temperature. *Acropora muricata* (ANOSIM: R = 0.633, *P* = 0.0005), *Cyphastrea chalcidicum* (ANOSIM: R = 0.782, *P* = 0.0006), *Coelastrea aspera* (ANOSIM: R = 0.687, *P* = 0.0002) and *Pachyseris rugosa* (ANOSIM: R = 0.613, *P* = 0.0026) (Table 3). In contrast to the inshore corals, however, there was a consistent significant decline in algal density, *F*_v_/*F*_m_^MT^, and ETR under higher temperature across all corals. Also, algal cell volume rose significantly for all offshore heated symbionts (Fig. 4A–D). Changes in other physiological metrics were typically unique for each coral species or, in some cases, shared among two coral species. For *Cladocopium 21* in heated *A*. *muricata*, carbohydrate content, PSII functional absorption cross section (σ_PSII_), τ_PQ_ and NPQ also increased (Fig. 4E, Table S7), while soluble protein, τ_PQ_ and σ_PSII_ increased with rising temperature in *C*. *40* in *P*. *rugosa* (Fig. 4B, Table S7). For *Cladocopium 3u* in *C*. *chalcidicum*, soluble protein, lipid and carbohydrate content, τ_PSII_, τ_PQ,_ σ_PSII_ and photosystem II connectivity (ρ) increased under elevated temperature. (Fig. 4E, Table S9). Lastly, soluble protein, lipid content, net photosynthesis cell^−1^, NPQ, and ρ increased in *Cladocopium 40* in offshore *C*. *aspera* (Fig. 4E, Table S10).

**Figure 5:**
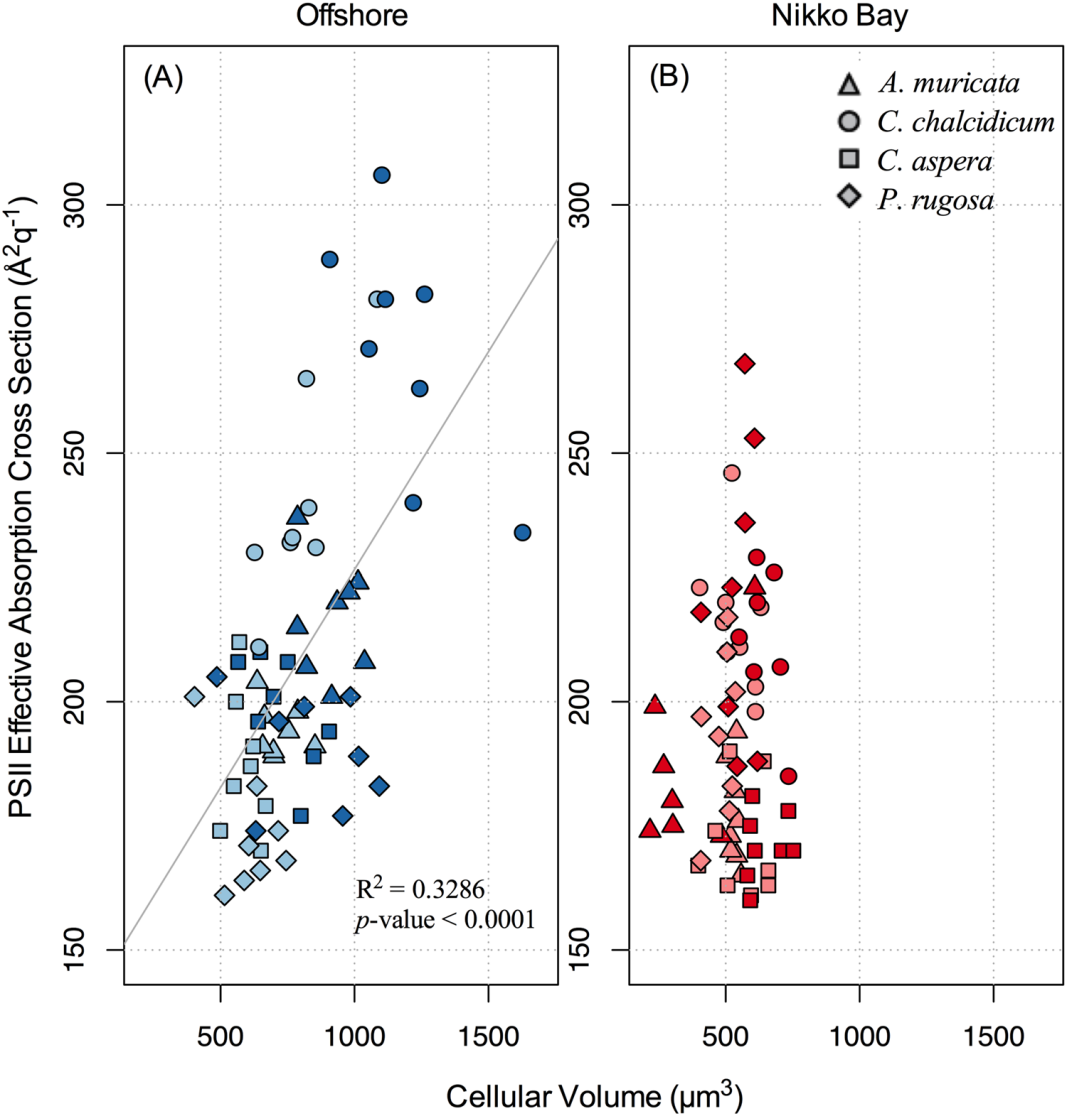
Regression plot between cellular volume (y-axis) and PSII effective absorption cross section (x-axis). The Left panel reflects the various Clade C (Blue) symbionts found at the Offshore site whereas the Right panel reflects the *D*. *trenchii* (Red) symbionts found in Nikko Bay. Light colors indicate (27.5 °C) and dark colors indicate (32 °C). Shapes depict the host coral species (triangle) *Acropora*, (circle) *Cyphastrea*, (square) *Coelastrea*, (diamond) *Pachyseris*.

When comparing symbionts from all corals at each site, there was a significant positive correlation (*P* < 0.0001) between symbiont volume and PSII functional absorption cross section (σ_PSII_) in the offshore corals. In particular, algal cell volume within heat treated corals tended to increase more relative to the rise in σ_PSII_ (Fig. 5A). In contrast, there was no consistent relationship between these variables across the control and heated corals from inshore habitats (Fig. 5B).

## Discussion

Pacific Corals with open modes of symbiont acquisition exhibit flexibility for certain host-generalist symbionts^40,46^. These host-symbiont combinations are determined by reef environments where new corals successfully recruit. Thus, entire Pacific coral communities are often dominated by one or a few host-generalist symbiont species. Consistent with this ecological dynamic, corals from inshore and offshore habitats, only 10 km apart, exhibited profound differences in their symbionts (Fig. 1). Most offshore colonies harbored just one of a variety of *Cladocopium* spp. depending on the coral species. Thus, while corals exhibit some degree of flexibility, they generally associate with a particular symbiont in a given environment^40,46^. The variation in *Cladocopium* spp. in our experimental corals indicates that there are significant differences in host-symbiont compatibility in offshore barrier reef environments.

The extreme environment of the inshore Rock Island habitats has reduced the possible symbionts in many corals to *Durusdinium trenchii*. *Durusdinium trenchii* is a host-scleractinian generalist whose populations are widespread and connected genetically throughout the Indo West Pacific^43,47^. Microsatellite genotyping of *D*. *trenchii* characterized numerous unique clones with each colony containing one dominant *D*. *trenchii* clone genotype; and thus consistent with previous findings that most coral colonies under ambient temperature conditions harbor just one symbiont genotype^48–51^. In some cases, closely related clones originated from the same host species. For example, *D*. *trenchii* from *Cyphastrea chalcidicum* were genetically similar to each other (Fig. 1D). However, when the *D*. *trenchii* genotypes from all four coral species were considered, only 9% of the overall variance could be attributed to host level differences, and further analyses of inshore corals from multiple locations around Palau show high homogeneity in the distribution of *D*. *trenchii* genotypes across these and other coral taxa (Suppl. Fig. 1). Thus there is high genotypic diversity of *D*. *trenchii* within inshore reefs but evidence is ambiguous to support the possibility of host specificity among similar clones.

Corals living in reef systems with a history of prolonged high temperature often display greater thermal tolerance^17,42,52–54^. Similarly, inshore colonies with *Durusdinium trenchii* fared measurably better than their offshore conspecifics harboring *Cladocopium* spp. (Fig. 2). The Rock Island reefs of Palau have garnered attention as they thrive in naturally warm and low pH (~7.9 ± 0.1) seawater^10,11,15^. The dominance of *D*. *trenchii* in these coral communities explains in large part why they persist and thrive under conditions that would eliminate most reef-building corals from other regions around the world (Fig. 1C)^8^. The substantial physiological disparities measured among colonies with different species of symbionts thus explains marked differences in bleaching severity across Palauan reef habitats^9,10^.

Although the distribution of *D*. *trenchii* genotypes was homogeneous and thus represented a single population, there were clear differences in their physiology across coral species (Table 3, Fig. 3A–D). For *D*. *trenchii physiology*, separation of ambient and elevated temperature samples was less than that observed across host species (Table 3). In addition, the direction of response to temperature differed across host species and, as depicted by the heat map (Fig. 3E), this likely reflects a unique response inherent to each host/symbiont combination (Fig. 3A–D). Together, these results suggest a strong host influence on *D*. *trenchii* physiology which leads to host dependent responses to elevated temperature (Fig. 3E). The two offshore corals harboring the same alga (*Cladocopium C40*) also revealed a strong host influence on symbiont physiology (Fig. 4C,D, Table 3) and helps to explain some differences in response to elevated temperature. For example, slower plastoquinone turnover (i.e. higher τ_PQ_), along with an increase in functional PSII absorption cross section, was characteristic of *C*. *40* symbionts in offshore *Pachyseris rugosa* in response to high temperature. In contrast, high temperatures induced an increase in net photosynthesis, NPQ, PSII connectivity, and lipids in *C*. *40* symbionts in *Coelastrea aspera*. Differences in host tissue thickness and pigmentation, along with skeletal structure may substantially influence the internal light fields of endosymbiotic algae^25,27,55,56^. Furthermore, host tissue biochemistry in the form of different metabolites and available nutrients may also alter symbiont physiology^57^. While beyond the immediate scope of our experimental design, such host attributes possibly contribute to the variability in *D*. *trenchii* and *Cladocopium C40* physiology observed here and are worthy of more detailed study.

Despite hosting thermally tolerant *Durusdinium trenchii*, inshore *Acropora muricata* and *Pachyseris rugosa* lost symbionts when exposed to high temperature (Fig. 3A,C). Significant *D*. *trenchii* loss has also been noted in Caribbean corals after two rounds of repeated experimental heating^58^ and recovery^45,58^. Importantly, the degree of loss tends to be less when compared with the same corals that are initially dominated by other species of symbionts^45,58^. Loss in *D*. *trenchii* and reduced *F*_v_/*F*_m_^MT^ in *P*. *rugosa*, was accompanied by lower ETR and increased NPQ, and τ_PQ_, all of which are common phenotypic proxies of photochemically stressed algae when monitored by active chlorophyll *a* fluorescence^2,4,59–61^. Unlike *A*. *muricata* and *P*. *rugosa*, algal density remained stable in heated *C*. *chalcidicum* and *C*. *aspera* despite marked differences in photochemistry exhibited by these coral species (Fig. 3B,C). In particular, both τPSII and τPQ time constants declined (i.e. transport rates increased) in *C*. *aspera*, while τPQ increased (i.e. rates slowed down) and total Chl *a* dropped within *D*. *trenchii* in *C*. *chalcidicum*. Large differences in reactive oxygen species (ROS) scavenging are prevalent across different coral species^62^ and such activity may have enabled the maintenance of symbiont number in *C*. *chalcidicum* and *C*. *aspera* despite some significant changes in photochemistry.

In contrast to *P*. *rugosa* and *C*. *chalcidicum*, the decline in Fv/Fm^MT^ was less pronounced for *D*. *trenchii* in *A*. *muricata* and was accompanied by increased cellular protein and carbohydrates. A similar temperature driven increase in symbiont biomass was noted in *D*. *trenchii* in *Turbinaria reniformis* and *Cladocopium C15* in *Montipora monasteriata* following heating^3^, and may be more indicative of thermal acclimation than stress. This is further supported by the significant increase in net cellular photosynthesis for remaining symbionts that accompanied algal loss in *A*. *muricata*. This paradoxical rise in photosynthesis as symbiont cell number declines may have been driven by alleviation of carbon limitation while *in hospite*^63,64^. Interestingly, heating led to a significant decline in *D*. *trenchii* cell volume in *A*. *muricata* yet Chl *a* cell^−1^ did not change. Stable pigment and declining cell size may have contributed to the increase in net photosynthesis in this coral. In contrast, following exposure to a combination of high temperature and CO_2_, *D*. *trenchii* cell volume increased in the coral *Turbinaria reniformis*, yet this was offset by changes in Chl *a* as well as photosynthesis^3,63^. However, *D*. *trenchii* volume did not change with heating in the other inshore corals, so this may reflect one of several strategies for acclimating to high temperature that is driven, in part, by how a particular host coral facilitates, or limits, transport of resources to the symbiont.

*Cladocopium* in offshore corals experienced greater stress at 32 °C, and the physiological responses among offshore corals were more similar, than corals of the same species with *Durusdinium trenchii*. The similar phenotype in physiological stress was revealed by greater correspondances in the directional shift in MDS response vectors after heating (Fig. 4), which was driven by significant loss in *F*_v_/*F*_m_^MT^, ETR and symbiont density, along with increased symbiont cell volume (Fig. 4A–E). Declining *F*_v_/*F*_m_^MT^, ETR, and symbiont density are now common proxies of coral bleaching. Despite an overall similar response to high temperature, some variability across offshore corals was observed. For example, connectivity among PSII reaction centers increased in heated *Cladocopium C40* (*Coelastrea aspera*) and *Cladocopium C3u (Cyphastrea chalcidicum*), potentially indicating greater transfer of excitation energy from damaged to remaining functional reaction centers. In some symbionts thermal photoinactivation may manifest downstream of the plastoquinone pool^65,66^, and declining electron transport out of the PSII reaction center and PQ pool (τ_PSII_ and τ_PQ_ respectively) in *Cladocopium C3u* symbionts suggests significant functional loss across multiple points of the photosynthetic electron transport chain. In contrast, non-photochemical quenching (NPQ) increased significantly in *Cladocopium* C*21* in *Acropora muricata* and *Cladocopium C40* in *Coelastrea aspera* (Fig. 4A,B). Enhanced NPQ is a common photoprotective mechanism, resulting in dissipation of excess excitation energy away from PSII reaction centers^1,67^ while electron transport out of PSII and the PQ pool did not change in *Cladocopium C21* (*A*. *muricata*) and *Cladocopium C40* (*Coelastrea aspera*) symbionts.

An important difference between heated inshore vs. offshore corals was the positive correlation between symbiont cell volume and PSII functional absorption cross section in the offshore corals (Fig. 5A). This pattern is in marked contrast to typical responses of light harvesting and PSII reaction center turnover and repair documented across a broad range of cell size in other phytoplankton. For example, as centric diatom cell volume increases, σ_PSII_ declines^68^. As cell volume increases, so too does Chl *a*, thereby leading to a rise in pigment packaging and a reduction in the efficiency of light harvesting for photochemistry^68^. Healthy symbionts *in vitro* also display a similar cell size to σ_PSII_ relationship to that of other phytoplankton^69^. While the PSII reaction center repair rate is typically slower for larger cells, a decline in σ_PSII_ leads to a lower cross-section for PSII photoinactivation^68^. However, the increase in cell volume accompanied by a higher σ_PSII_ with heating shown here indicates that many of the offshore symbionts were unable to counterbalance the continued thermal photoinactivation by reducing the target area for photochemistry. Further, while increased volume in heated offshore symbionts may also reflect a release of resource limitation (e.g., carbon-limitation described above), higher temperatures will lead to a greater metabolic demand for cellular maintenance and repair such that increased cell size most likely exacerbates the negative effects of heating in offshore habitats, but not inshore habitats (Fig. 5B). Alternatively, host-centric influences may also be considered, including a loss in controlling symbiont cell division which may lead to an increase in symbiont size.

In summary, we observed four distinct physiological responses to elevated temperature exhibited by a single symbiont species, *Durusdinium trenchii* in different hosts. Even within a thermally tolerant population of symbionts, host dependent differences may create significant variance in symbiont physiology and alter the overall response of particular host-symbiont combinations to elevated temperature. Host physiological differences likely affected the state of symbiont thermal sensitivity and further highlights the importance of the host environment in modulating the symbiont’s thermal response. Importantly, data from a broad selection of physiological proxies are needed to fully encapsulate differences within and between distantly and closely related symbionts. These multivariate comparisons provide a more in-depth understanding of the unique thermal response expressed by a particular host–symbiont combination. Moreover, this detailed view better identifies particular host/symbiont combinations that are most resilient to periodic episodes of high-temperature and are potentially able to endure increasing ocean warming into the future.

## Materials and Methods

### Coral collection

Offshore corals were collected from Rebotel reef (7°14.93′N, 134°14.149′E) and inshore corals were collected within Nikko Bay (7°32.48′N, 134° 49.34′E). The corals *Acropora muricata* (branching) and *Coelastrea aspera* (massive) were sampled in March of 2014 from both sites and subsequently used in an initial thermal experiment. In March of 2015 two other coral species, *Pachyseris rugosa* (plating/encrusting) and *Cyphastrea chalcidicum* (encrusting) were sampled and treated in a similar manner. A total of 8 colonies of each species were collected at each site at a depth between 5–10 meters (offshore) or 1–5 meters (Inshore) and at least 10 meters apart. Differences in collection depth were necessary due to the natural distribution of these species at each site and in order to ensure all colonies were collected from similar light conditions (maximal mid-day *in situ* light of 800–1000-μmol quanta m^−2^ s^−1^). Colonies were transported back to the Palau International Coral Research Center (PICRC) and fragmented into five replicate nubbins and placed into a 1200 L flow-through aquarium and held at 27.5 °C. Seawater was collected directly off of a nearby pier at a depth of 3 m and then passed through a pressurized sand filter and aquarium filter pads prior to use in flow-through and experimental treatment systems. Coral nubbins were attached to 2-inch square PVC tiles with marine epoxy (Splash zone compound A-788) and held at ambient conditions in flow-through bins as described above for 12–16 days prior to the start of the experiment. Control and experimental bins (see below) were maintained outdoors underneath clear plastic film (Sun Selector, Ginegar Plastic Products) to block periodic rainfall and a 60% shade cloth providing a peak midday light intensity of 800 μmol quanta m^−2^ s^−1^, as measured with a light sensor (LiCor LI-192).

### Experimental system

Each treatment system consisted of 7–12 (56 L) plastic treatment bins connected to a central (~1200 L) sump. Seawater within each sump was set to either heated or ambient temperature conditions prior to being sent to the treatment bins. Ambient temperature was maintained via a chiller system and a series of titanium heating elements were used for high temperature treatments. For each treatment, three replicate fragments from each colony were placed within separate treatment bins. For the heated treatment, the temperature was gradually ramped from 27.5 °C to 32 °C over 4 days, and then maintained at 32 °C for an additional 10 days for a total of 14 days of heating. Temperature within the control treatment was maintained at 27.5 °C throughout the 14-day experiment. Treatment bins and PVC tiles were cleaned every other day to prevent algal fouling, and coral fragments were rotated within their respective bins every other day to ensure a uniform light exposure and minimize possible tank effects.

At the start of the experiment (day 0), one fragment from each coral colony was removed from control and treatment tanks and processed for symbiont photo-physiology and biomass metrics (described below). Additional fragments were then sampled on days 9 (4 days of temperature ramping + 5 days at 32 °C) and 14 (4 days of temperature ramping and 10 days at 32 °C). Coral tissue was removed by airbrush (100 psi) with filtered (0.22 μm) seawater. The resulting slurry was homogenized with a Tissue Tearor (BioSpec products, Inc), and then divided into 2 mL aliquots. One aliquot was preserved with 1% glutaraldehyde for cell enumeration and stored at 4 °C. All other aliquots were centrifuged for 2 minutes (5,000 × *g*) and the supernatant was discarded. Algal subsamples from each colony were suspended in DNA preservation buffer (Seutin *et al*. 1991) and stored at 4 °C. The remaining algal samples were immediately frozen (−20 °C) and shipped back to the United States and stored at −20 °C until further analyses.

### Symbiont density and volume

Algal density and volume were measured by microscope. Replicate cell counts (n = 4–6) were performed for each coral sample with a hemocytometer and photographed with an EVOS digital fluorescent microscope fitted with a calibrated micrometer. Digital photos were analyzed in Image J (NIH), with the Analyze Particles function using methods similar to^69^. Colony surface area was determined by the foil method^70^ for *C*. *aspera*, *P*. *rugosa*, and *C*. *chalcidicum*, whereas the hot wax method^71^ was used for the branching *A*. *muricata*.

### Algal biochemical composition and photopigment concentration

For analysis of soluble proteins and carbohydrates, algal cells were lysed with glass beads in 2 mL of filtered seawater in a bead-beater (BioSpec products, Inc) for 2 minutes. 50 μL of the lysate was used for protein quantification by the BCA method (Thermo Scientific Pierce), with a bovine serum albumin standard^72^. 100 μL of the lysate was used for carbohydrate quantification by the sulfuric acid/phenol method^73^, with d-glucose as standard. For lipid extraction, pelleted algae were freeze-dried overnight and then extracted in a chloroform:methanol:sodium chloride mixture (2:1:0.8)^74,75^. Total lipids were measured by a sulfo-phospho-vanillin colorimetric assay using corn oil as a standard^76^. Absorbance measurements for lipid, carbohydrate and protein assays were made at 540, 485 and 595 nm respectively. For determination of chlorophyll *a*, algal pellets were resuspended in 90% methanol and then homogenized by bead beating for two minutes. Samples were incubated at −20 °C for two hours, centrifuged at 2300-*g* and supernatant absorbance was recorded at 665, 652 and 750-nm and chlorophyll *a* calculated by published equations^77^. All absorbance measurements were determined using a FLUOstar Omega plate reader (BMG Labtech, Germany). Protein, lipid, carbohydrate and chlorophyll data were normalized to algal cell number. For each assay, two technical replicates were run for each sample replicate.

### Symbiont photophysiology

Maximal net coral photosynthetic rates (P_max_) and light acclimated dark respiration (R_L_) were measured via oxygen evolution in custom clear acrylic chambers (300 mL) fitted with a stir bar and a fiber optic oxygen probe connected to a Fibox 4 fiber optic oxygen system (PreSens). Chambers were held in a water bath to maintain the control and experimental temperature. Illumination was supplied by a custom 24 LED array (Cree Cool White XP-G R5) set to a light intensity of 500-μmol quanta m^−2^ s^−1^. At this light setting, corals reached maximum net photosynthesis (algal cell^−1^) and showed no signs of photoinhibition (data not shown). P_max_ was recorded for 15–20 minutes, followed by a 15-minute dark incubation after the lights were switched off to record the light acclimated dark respiration (R_L_). Gross photosynthetic rates (P_gross_) were then calculated as (P_max_ − R_L_).

Active chlorophyll *a* fluorescence of each coral fragment was recorded by a Fluorescence Induction and Relaxation (FIRe) fluorometer (Satlantic Inc., Halifax) fitted with a fiber optic LED excitation light source (peak λ 455 nm)^2^. On the final day of the experiment, measurements were taken after thirty minutes of dark acclimation at midday. Each measurement consisted of five iterations of a 100-*μ*s single turnover flash, followed by a 2000-*μ*s relaxation phase consisting of 1-*μs* light flashes spaced 59-*μs* apart. This was followed by a 100-ms multi-turnover flash and relaxation phase. The single turnover saturation pulse reduces the primary electron acceptor (Q_A_) of the photosystem II (PSII) reaction center one time, whereas the longer multi-turnover saturation flash reduces both primary and secondary acceptors within the PSII reaction center and the plastoquinone (PQ) pool^78^. All photochemical parameters recorded by the FIRe fluorometer were calculated by fitting each fluorescence transient curve in *FIREPRO* software^79^. The maximum PSII quantum yield was calculated as *F*_v_/*F*_m_ = (*F*_m_-*F*_o_/*F*_m_), Where *F*_o_ and *F*_m_ are the minimum and maximum fluorescence after dark acclimation respectively. Maximal fluorescence recorded in the light acclimated state is abbreviated as *F*_m_′, and maximal fluorescence yield (dark or light acclimated) by single and multi-turnover flashes are designated as such by ^ST^ or ^MT^ notation respectively. The kinetics of fluorescence relaxation after single and multi-turnover flashes (abbreviated as τ_PSII_ and τ_PQ_ respectively) were used to calculate the reoxidation rates of the Q_A_ acceptor and the PQ-pool respectively and provide a better understanding of possible break points in the photosynthetic electron transport chain.

Electron transport rate and non-photochemical quenching were calculated after a five minute incubation under a 500-μmol quanta m^−2^ s^−1^ white light source (RG5-cool white CREE LED), and calculated as,

$${{\rm{ETR}}}^{{\rm{RCII}}}({\rm{mol}}\,{{\rm{e}}}^{-}{\rm{mol}}\,{{\rm{RCII}}}^{-1}{{\rm{h}}}^{-1})={\rm{PFD}}\times {F}_{{\rm{q}}}^{^{\prime} }/{F}_{{\rm{m}}}^{^{\prime} {\rm{ST}}}\times {{\rm{\sigma }}}_{{\rm{PSII}}}\times 21.683$$

where PFD is the photon flux density, *F*_q_′/*F*_m_′^ST^ is the operating efficiency of PSII in the light acclimated state, σ_PSII_ is the PSII functional absorption cross section in the dark, and 21.683 converts seconds to hours, *µ*mol e^−^ to mol e^−^ and Å^2^ quanta^−1^ to m^2^ mol RCII^−1^ ^80,81^. Incorporating σ_PSII_ into the ETR calculation is more accurate than the conventional “relative” ETR calculated in many studies utilizing rapid light curve methods, often recorded by PAM fluorescence, that do not account for any changes in light absorption or functional cross section^82^, however there are still limitations in quantifying ETR and functional absorption cross section in corals^82^. In particular, ETR will tend to be overestimated when relying on the incident irradiance as the PFD in ETR calculations, rather than the internal scalar irradiance, which requires more sophisticated and labor intensive measurements of the internal light field within a coral polyp^83^. Coral skeletons contribute to substantial photon path length enhancement and higher scalar irradiance, while the coral tissue may also attenuate such light^24,84^ and both factors can markedly affect σ_PSII._ In addition, σ_PSII_ is a wavelength dependent variable that tends to be lower when comparing values from *in hospite* algae against those collected from optically thin algal cultures^83,85^. Nevertheless, when measured in the blue spectrum, recent evidence shows that the difference in σ_PSII_ (λ) between symbionts *in hospite* and in culture are closely correlated and largely dependent on light availability.

Non photochemical quenching (NPQ) was calculated as,

$${\rm{NPQ}}({\rm{dimensionless}})=({F}_{{\rm{m}}}^{{\rm{MT}}}\mbox{--}{F}_{{\rm{m}}}^{^{\prime} {\rm{MT}}})/{F}_{{\rm{m}}}^{^{\prime} {\rm{MT}}}$$

A pulse amplitude modulation fluorometer (Diving PAM, Waltz, Germany) was also used to measure *F*_v_/*F*_m_ on a daily basis. Fragments were sampled one hour after sunset in three separate locations using a 0.6-second saturation pulse (saturation intensity > 8000-μmol quanta m^−2^ s^−1^). *F*_v_/*F*_m_ samples were then averaged together in order to calculate the mean *F*_v_/*F*_m_ for each fragment. All photo-physiological parameters measured are also defined in Table 1.

### Symbiont identification and phylogenetic analysis

Symbiont identity was verified through amplification and sequencing of rDNA. The internal transcribed spacer 2 region (ITS2) was analyzed using denaturing gradient gel electrophoresis to identify the sequence from the dominant variant in the ribosomal array; which is generally diagnostic of the resident symbiont species^86,87^. See previously published protocols for DGGE screening of ITS2 rDNA and sequencing of the dominant variants^87,88^. Briefly, the ITS2 region was amplified and PCR products were electrophoresed on denaturing gradient gels (45–80%) for 1500 volt-hours at 60 °C. Diagnostic bands were excised from the gel and re-amplified prior to sequencing on an ABI PRISM 3100 Genetic Analyser.

The dinoflagellate family Symbiodiniaceae has recently undergone taxonomic revision, and the previous clades of *Symbiodinium* common to reef-building corals designated as B, C, and D are now assigned to new genera^89^. In keeping with the current nomenclature for ITS2 identification, symbionts that are not characterized to species are listed here by the new genera and the previous ITS2 number-letter designation. Sequences of the large ribosomal sub-unit were also obtained via direct sequencing using the primers and amplification settings specified in^90^. Fourteen microsatellite loci previously developed for *Durusdinium*^91,92^ were used to determine the strain diversity of *D*. *trenchii* within and between colonies. An analysis of molecular variance (AMOVA) was performed on the microsatellite sizes of the multilocus genotypes of *D*. *trenchii* using the program GenAIEx^93^. Results were also visualized using a Principal Coordinates analysis using the vegan package in R. Figure 1D reflects only colonies used within the experiment whereas additional colonies (collected in 2009) are included in the Supplemental Fig. 1 and provide a broader overview of *D*. *trenchii* genotypes in Nikko Bay.

### Statistical analysis of symbiont physiology

Bleaching was first assessed by comparing changes in algal density and *F*_v_/*F*_m_^MT^ on days 9 and 14 between ambient and heated samples by an Analyses of Similarity test (ANOSIM), with 9,999 permutations. When significant separation was observed, each variable was compared by a Wilcoxon *t*-test.

When the same symbiont species (as identified by rDNA sequencing) was found in multiple coral species, host dependent differences in symbiont physiology were compared by ANOSIM analysis using 14 physiological variables collected on the final day. These variables included *F*_v_/*F*_m_^ST^, PSII connectivity (ρ), functional absorption cross section (σ_PSII_), non-photohemical quenching (NPQ), electron transport rate (ETR), PSII reaction center turnover (τ_PSII_), Plastoquinone pool turnover (τ_PQ_), net photosynthesis, symbiont number, lipid, carbohydrate, protein, volume and chlorophyll *a* content. Only colonies maintained at the control temperature were included in the analysis and all variables were standardized to remove potential error from differences in variance across physiological measurements. Non-metric multidimensional scaling of all 14 physiological variables (after log(x + 1) transformation^94^) was used in order to visualize separation across different treatments and coral species for each site.

All data were tested for homogeneity of variance and normality using the Shapiro-Wilks tests. If either test was significant (*P* < 0.05), data was log transformed and then retested. An ANOSIM analysis was used within each host/symbiont combination in order to test for overall response to elevated temperature. Each variable was also individually tested via a t-test. If the data failed to meet normality, a non-parametric Wilxocon test was used instead. The resulting significant differences in physiological parameters were graphically represented in a heat map as the average log_2_ fold change for each host/symbiont combination. The mean and standard error for each variable are also provided for each host symbiont combination in Supplementary Tables 1 and 2). All statistical analyses were performed using R software (R Core Team, 2019) with the ‘vegan’, ‘car’, ‘edgeR’, ‘gplots’, ‘fmsb’ and ‘pgirmess’ packages installed. Data and R scripts for generation of Figs 3–5 have been deposited online via Github (khoadley/Scientific-Reports2019).

## Supplementary information

Supplementary Information

## Data Availability

The datasets generated during and/or analyzed during the current study are available from the corresponding author on reasonable request. Data and R scripts for Figs 3–5 are available via github (khoadley/Scientific-Reports2019).
