# Supplementary material for: Host–symbiont combinations dictate the photo-physiological response of reef-building corals to thermal stress
Source: Sci Rep. 2019 Jul 10;9:9985. doi: 10.1038/s41598-019-46412-4 (PMC6620294; doi:10.1038/s41598-019-46412-4)
Supplement: Supplementary file 1 — Supplementary Information [file 41598_2019_46412_MOESM1_ESM.docx]

**Title:** Host–symbiont combinations dictate the photo-physiological response of reef-building corals to thermal stress

**Author list**

Kenneth D. Hoadley^1+^, Allison M. Lewis^2^, Drew C. Wham^2^, D. Tye Pettay^1^, Chris Grasso^1^, Robin Smith^4^, Dustin W. Kemp^3^, Todd C. LaJeunesse^2^ and Mark E. Warner^1^

**Author affiliations**

^1^ School of Marine Science and Policy, University of Delaware, Lewes, DE, United States

^2^ Department of Biology, Pennsylvania State University, University park, PA, United States

^3^ Department of Biology, University of Alabama at Birmingham, AL, United States

^4^ Science Under Sail, United States

^+^ Present Address: GEOMAR Helmholtz Centre for Ocean Research, Kiel, United Germany

**Corresponding Author:**

Kenneth D. Hoadley, khoadley@geomar.de

**Supplementary figure 1:** Similar to Figure 2, a principle coordinates plot utilizing 14 microsatellite markers to visualize variance across *S. trenchii* symbionts for all four coral species in Nikko bay. Coral species are separated by color. Triangles reflect samples from the experiment (2014) whereas circles reflect additional rock island coral colonies sampled in 2009. The additional samples reflect a greater variance for *S. trenchii* genotypes in *Cyphastrea* than is observed for experimental colonies (2014) alone.

**Supplementary table 1:** Mean +/- standard deviation for each variable at ambient and elevated temperatures for *S. trenchii.* C = ambient temperature, T = elevated temperature. Units are in table 1 of manuscript.

|  | Treatment | C | T | C | T | C | T | C | T |
| --- | --- | --- | --- | --- | --- | --- | --- | --- | --- |
|  | Host | Acropora | Acropora | Cyphastrea | Cyphastrea | Coelastrea | Coelastrea | Pachyseris | Pachyseris |
| Protein | mean | 4.38E-04 | 9.78E-04 | 3.32E-04 | 2.87E-04 | 5.01E-04 | 9.22E-04 | 4.32E-04 | 6.03E-04 |
|  | se | 6.09E-05 | 1.38E-04 | 2.35E-05 | 2.88E-05 | 1.25E-04 | 1.98E-04 | 2.16E-05 | 9.99E-05 |
| Carbohydrate | mean | 5.07E-03 | 1.12E-02 | 1.47E-03 | 1.23E-03 | 1.18E-03 | 1.85E-03 | 1.54E-03 | 2.54E-03 |
|  | se | 5.40E-04 | 1.42E-03 | 1.71E-04 | 1.13E-04 | 1.40E-04 | 2.73E-04 | 1.23E-04 | 8.66E-04 |
| Lipid | mean | 6.88E-04 | 1.01E-03 | 5.75E-04 | 3.78E-04 | 2.69E-03 | 2.13E-03 | 1.96E-04 | 4.63E-04 |
|  | se | 1.65E-04 | 3.26E-04 | 7.47E-05 | 8.07E-05 | 5.67E-04 | 6.40E-04 | 4.72E-05 | 1.18E-04 |
| Volume | mean | 532.649915 | 301.433058 | 559.826372 | 626.746448 | 547.677781 | 645.612531 | 479.369178 | 562.488109 |
|  | se | 6.194123 | 39.013881 | 21.793578 | 27.301507 | 44.005254 | 25.662511 | 16.758783 | 16.935468 |
| Chlorophyll | mean | 6.7706082 | 3.916769 | 4.230956 | 3.169302 | 3.8112923 | 5.7261758 | 2.8262551 | 3.5076916 |
|  | se | 0.658313 | 2.6941479 | 0.2467076 | 0.2864586 | 0.4398935 | 0.7851855 | 0.2308363 | 0.5545985 |
| Photosynthesis | mean | 3.53E-09 | 8.28E-09 | 2.14E-09 | 1.54E-09 | 7.72E-09 | 1.15E-08 | 1.52E-09 | 1.61E-09 |
|  | se | 5.57E-10 | 1.31E-09 | 1.80E-10 | 2.06E-10 | 2.51E-09 | 2.67E-09 | 1.51E-10 | 2.67E-10 |
| Sigma | mean | 177.25 | 181.333333 | 216.142857 | 212 | 170.166667 | 171.125 | 191 | 214.777778 |
|  | se | 3.614208 | 4.12041 | 5.861206 | 4.898979 | 4.362084 | 2.408597 | 5.734884 | 10.741635 |
| Connectivity | mean | 0.13625 | 0.14 | 0.045714286 | 0.05125 | 0.113333333 | 0.12875 | 0.055 | 0.055555556 |
|  | se | 0.026520713 | 0.014832397 | 0.002020305 | 0.00125 | 0.013333333 | 0.013152607 | 0.002687419 | 0.002421611 |
| ETR | mean | 358078.483 | 318917.178 | 456600.614 | 319356.71 | 267183.347 | 317773.851 | 275679.83 | 166633.855 |
|  | se | 39539.018 | 41361.247 | 49737.214 | 32655.907 | 28764.607 | 24035.61 | 19376.391 | 7306.771 |
| Density | mean | 369829.682 | 169403.495 | 820014.301 | 984558.026 | 386259.153 | 314722.407 | 1056538.062 | 545490.323 |
|  | se | 36949.092 | 36811.568 | 67449.94 | 103966.736 | 58531.106 | 53419.488 | 68286.365 | 78450.995 |
| tPSII | mean | 1458.875 | 1604.66667 | 1696.28571 | 1589.125 | 2082.83333 | 1815 | 1741.8 | 1707.66667 |
|  | se | 74.55042 | 29.55522 | 66.66609 | 60.15083 | 84.2304 | 71.13518 | 58.37347 | 91.02106 |
| tPQ | mean | 19832.375 | 19555.6667 | 23646.5714 | 27722.25 | 18725 | 13648.625 | 26327.5 | 31496.1111 |
|  | se | 855.2894 | 943.2985 | 650.3123 | 881.5108 | 2256.7817 | 894.1945 | 838.4186 | 1021.7727 |
| FvFm^MT^ | mean | 0.542375 | 0.514666667 | 0.544448277 | 0.507207053 | 0.535666667 | 0.511875 | 0.542124012 | 0.433809343 |
|  | se | 0.005766088 | 0.003527668 | 0.008514625 | 0.010739646 | 0.0042947 | 0.008580371 | 0.005843058 | 0.013953458 |
| NPQ | mean | 0.2125294 | 0.21075427 | 0.54660364 | 0.8010928 | 0.31398485 | 0.22347768 | 0.61763778 | 0.89477692 |
|  | se | 0.04878823 | 0.05895733 | 0.09461401 | 0.11364252 | 0.05889106 | 0.03231361 | 0.04992958 | 0.06788581 |

**Supplementary table 2:** Mean +/- standard deviation for each variable at ambient and elevated temperatures for clade C symbionts offshore*.* C = ambient temperature, T = elevated temperature. Units are in table 1 of manuscript.

|  | Treatment | C | T | C | T | C | T | C | T |
| --- | --- | --- | --- | --- | --- | --- | --- | --- | --- |
|  | Host | Acropora (C21) | Acropora (C21) | Pachyseris (C40) | Pachyseris (C40) | Cyphastrea (C3u) | Cyphastrea (C3u) | Coelastrea (C40) | Coelastrea (C40) |
| Protein | mean | 4.63E-04 | 7.90E-04 | 4.75E-04 | 9.37E-04 | 5.05E-04 | 1.08E-03 | 3.93E-04 | 7.23E-04 |
|  | se | 7.69E-05 | 1.38E-04 | 4.73E-05 | 1.92E-04 | 7.81E-05 | 1.79E-04 | 5.08E-05 | 1.15E-04 |
| Carbohydrate | mean | 3.37E-03 | 7.17E-03 | 1.73E-03 | 2.19E-03 | 1.98E-03 | 4.14E-03 | 1.86E-03 | 2.86E-03 |
|  | se | 1.75E-04 | 7.42E-04 | 3.59E-04 | 4.05E-04 | 2.95E-04 | 6.95E-04 | 1.12E-04 | 4.95E-04 |
| Lipid | mean | 9.68E-04 | 1.95E-03 | 3.69E-04 | 8.51E-04 | 7.05E-04 | 1.23E-03 | 9.47E-04 | 1.96E-03 |
|  | se | 1.52E-04 | 5.14E-04 | 8.35E-05 | 3.14E-04 | 1.16E-04 | 1.52E-04 | 1.05E-04 | 3.45E-04 |
| Volume | mean | 718.658807 | 909.433327 | 656.123896 | 929.854279 | 802.916376 | 1210.981082 | 590.965171 | 731.846888 |
|  | se | 26.149945 | 35.519481 | 25.166821 | 56.609748 | 57.982399 | 83.133398 | 20.186894 | 40.604243 |
| Chlorophyll | mean | 6.8898058 | 7.825188 | 3.3020107 | 3.6467637 | 5.5316689 | 5.0508602 | 6.7794856 | 5.4096166 |
|  | se | 0.3244097 | 0.8484077 | 0.3278642 | 0.6572546 | 0.8932532 | 0.1974852 | 0.6376878 | 0.8971662 |
| Photosynthesis | mean | 2.83E-09 | 5.48E-09 | 1.48E-09 | 1.84E-09 | 3.33E-09 | 3.05E-09 | 1.43E-09 | 3.98E-09 |
|  | se | 7.59E-10 | 1.04E-09 | 2.54E-10 | 5.77E-10 | 6.87E-10 | 3.17E-10 | 3.71E-10 | 6.12E-10 |
| Sigma | mean | 194.25 | 216.75 | 171 | 190.833333 | 241.285714 | 270.714286 | 187 | 197.875 |
|  | se | 1.810189 | 4.06092 | 2.804758 | 3.885157 | 8.972748 | 9.956367 | 4.906264 | 3.997488 |
| Connectivity | mean | 0.19375 | 0.20875 | 0.063333333 | 0.105 | 0.041428571 | 0.052857143 | 0.095 | 0.2625 |
|  | se | 0.015916916 | 0.02614776 | 0.00421637 | 0.022323381 | 0.001428571 | 0.003595159 | 0.005 | 0.038114021 |
| ETR | mean | 452750.526 | 301376.083 | 292308.523 | 165016.665 | 458978 | 168424.251 | 385712.111 | 151821.656 |
|  | se | 27067.336 | 31117.351 | 26268.212 | 32861.872 | 40631.174 | 22518.494 | 30851.784 | 37849.988 |
| Cell Density | mean | 328661.857 | 207639.507 | 907664.734 | 479197.845 | 716127.545 | 289704.52 | 778319.449 | 386863.979 |
|  | se | 35865.069 | 32884.381 | 132357.845 | 117133.311 | 216385.723 | 44952.877 | 46523.953 | 53240.157 |
| tPSII | mean | 1136.625 | 1241.625 | 1600.33333 | 1724 | 1606.85714 | 1957.42857 | 1789 | 1989.125 |
|  | se | 82.39472 | 63.82368 | 152.35981 | 53.8467 | 70.03959 | 114.13419 | 126.535 | 107.99263 |
| tPQ | mean | 22128.375 | 25599.75 | 24242.5 | 31842.3333 | 20593.2857 | 26626.8571 | 18972.875 | 16811.125 |
|  | se | 742.5809 | 1449.2449 | 772.4145 | 2024.8196 | 556.7263 | 1006.7149 | 834.8579 | 1476.3678 |
| FvFm^MT^ | mean | 0.533125 | 0.457125 | 0.572316706 | 0.314567567 | 0.530071587 | 0.287512785 | 0.50525 | 0.372625 |
|  | se | 0.005022938 | 0.011534788 | 0.011385506 | 0.033558128 | 0.013892644 | 0.017683526 | 0.012829584 | 0.018752083 |
| NPQ | mean | 0.23983936 | 0.37053775 | 0.72433816 | 0.75425246 | 0.7808828 | 1.11932527 | 0.33504461 | 0.58325209 |
|  | se | 0.02689101 | 0.04270516 | 0.06633545 | 0.17356184 | 0.13773317 | 0.07870627 | 0.0469688 | 0.0851032 |

**Supplemental Table 3: *Acropora muricata* with *D. trenchii.*** Results from t-test. Variables with *P-values* less than *P* <0.05 **(boldface)** are visualized in the heatmaps.

| Variable | P-value |
| --- | --- |
| **FvFm** | **0.0017** |
| ETR | 0.5073 |
| NPQ | 0.9819 |
| Sigma | 0.4719 |
| P | 0.9041 |
| tPSII | 0.8319 |
| tPQ | 0.1022 |
| Chla | 0.3459 |
| **Photo** | **0.0089** |
| **Proteins** | **0.0127** |
| Lipids | 0.4908 |
| **Carbs** | **0.0059** |
| **Volume** | **0.0007** |
| **Density** | **0.0025** |

**Supplemental Table 4: *Pachyseris rugosa* with *D. trenchii.*** Results t-test. Variables with *P-values* less than *P* <0.05 **(boldface)** are visualized in the heatmaps.

| Variable | P-value |
| --- | --- |
| **FvFm** | **0.0001** |
| **ETR** | **0.0006** |
| **NPQ** | **0.0197** |
| Sigma | 0.0644 |
| P | 0.6754 |
| **tPSII** | **0.0026** |
| tPQ | 0.9126 |
| Chla | 0.5524 |
| Photo | 0.2319 |
| Proteins | 0.9551 |
| **Lipids** | **0.0289** |
| Carbs | 0.8665 |
| **Volume** | **0.0054** |
| **Density** | **0.0006** |

**Supplemental Table 5: *Coelastrea aspera* with *D. trenchii.*** Results from t-test. Variables with *P-values* less than *P* <0.05 **(boldface)** are visualized in the heatmaps.

| Variable | P-value |
| --- | --- |
| **FvFm** | **0.0324** |
| ETR | 0.2050 |
| NPQ | 0.2150 |
| Sigma | 0.8523 |
| P | 0.4270 |
| **tPSII** | **0.0438** |
| **tPQ** | **0.0339** |
| Chla | 0.0577 |
| Photo | 0.8518 |
| Proteins | 0.0996 |
| Lipids | 0.3450 |
| Carbs | 0.0525 |
| Volume | 0.0895 |
| Density | 0.3856 |

**Supplemental Table 6: *Cyphastrea chalcidicum* with *D. trenchii.*** Results from t-test. Variables with *P-values* less than *P* <0.05 **(boldface)** are visualized in the heatmaps.

| Variable | P-value |
| --- | --- |
| **FvFm** | **0.0179** |
| **ETR** | **0.0424** |
| NPQ | 0.1092 |
| Sigma | 0.5974 |
| **P** | **0.0437** |
| **tPSII** | **0.0028** |
| tPQ | 0.2547 |
| **Chla** | **0.0149** |
| Photo | 0.1148 |
| Proteins | 0.2516 |
| Lipids | 0.0969 |
| Carbs | 0.2648 |
| Volume | 0.0781 |
| Density | 0.2096 |

**Supplemental Table 7: *Acropora muricata* with *C. 21.*** Results from t-test. Variables with *P-values* less than *P* <0.05 **(boldface)** are visualized in the heatmaps.

| Variable | P-value |
| --- | --- |
| **FvFm** | **0.0006** |
| **ETR** | **0.0002** |
| **NPQ** | **0.0180** |
| **Sigma** | **0.0021** |
| P | 0.4592 |
| **tPSII** | **0.0297** |
| tPQ | 0.2654 |
| Chla | 0.5846 |
| Photo | 0.3495 |
| Proteins | 0.0721 |
| Lipids | 0.1206 |
| **Carbs** | **0.0006** |
| **Volume** | **0.0006** |
| **Density** | **0.0499** |

**Supplemental Table 8: *Pachyseris* *rugosa* with *C. 40****.* Results from t-test. Variables with *P-values* less than *P* <0.05 **(boldface)** are visualized in the heatmaps.

| Variable | P-value |
| --- | --- |
| **FvFm** | **0.0003** |
| **ETR** | **0.0134** |
| NPQ | 0.8770 |
| **Sigma** | **0.0025** |
| P | 0.0841 |
| **tPSII** | **0.0114** |
| tPQ | 0.4721 |
| Chla | 0.6524 |
| Photo | 0.3095 |
| **Proteins** | **0.0411** |
| Lipids | 0.1797 |
| Carbs | 0.4163 |
| **Volume** | **0.0032** |
| **Density** | **0.0361** |

**Supplemental Table 9: *Cyphastrea chalcidicum* with *C. 3u.*** Results from t-test. Variables with *P-values* less than *P* <0.05 **(boldface)** are visualized in the heatmaps.

| Variable | P-value |
| --- | --- |
| **FvFm** | **0.0006** |
| **ETR** | **0.0001** |
| NPQ | 0.0600 |
| **Sigma** | **0.0487** |
| **P** | **0.0117** |
| **tPSII** | **0.0005** |
| **tPQ** | **0.0258** |
| Chla | 0.2086 |
| Photo | 0.4695 |
| **Proteins** | **0.0188** |
| **Lipids** | **0.0184** |
| **Carbs** | **0.0070** |
| **Volume** | **0.0021** |
| **Density** | **0.0210** |

**Supplemental Table 10: *Coelastrea aspera* with *C. 40****.* Results from t-test. Variables with *P-values* less than *P* <0.05 **(boldface)** are visualized in the heatmaps.

| Variable | P-value |
| --- | --- |
| **FvFm** | **0.0001** |
| **ETR** | **0.0003** |
| **NPQ** | **0.0270** |
| Sigma | 0.1086 |
| **P** | **0.0009** |
| tPSII | 0.2286 |
| tPQ | 0.2494 |
| Chla | 0.2359 |
| **Photo** | **0.0217** |
| **Proteins** | **0.0148** |
| **Lipids** | **0.0104** |
| Carbs | 0.1949 |
| **Volume** | **0.0108** |
| **Density** | **0.0001** |
